# Lower Expression of MicroRNA-155 Contributes to Dysfunction of Natural Killer Cells in Patients with Chronic Hepatitis B

**DOI:** 10.3389/fimmu.2017.01173

**Published:** 2017-09-22

**Authors:** Jun Ge, Zuxiong Huang, Hongyan Liu, Jiehua Chen, Zhanglian Xie, Zide Chen, Jie Peng, Jian Sun, Jinlin Hou, Xiaoyong Zhang

**Affiliations:** ^1^State Key Laboratory of Organ Failure Research, Guangdong Provincial Key Laboratory of Viral Hepatitis Research, Department of Infectious Diseases, Nanfang Hospital, Southern Medical University, Guangzhou, China; ^2^Department of Hepatology, Mengchao Haptobiliary Hospital of Fujian Medical University, Fuzhou, China; ^3^Department of Hepatology, Affiliated Infectious Disease Hospital of Fujian Medical University, Fuzhou, China

**Keywords:** chronic hepatitis B, miR-155, natural killer cells, suppressor of cytokine signaling 1, telbivudine

## Abstract

MicroRNAs have been reported to be regulated in different ways in a variety of liver diseases. As a key modulator of cellular function in both innate and adaptive immunity, the role of miR-155 in chronic hepatitis B virus infection remains largely unknown. Here, we investigated the expression and function of miR-155 in chronic hepatitis B (CHB) patients. It was found that miR-155 expression in peripheral blood mononuclear cells (PBMCs) was lower in CHB patients than healthy controls (HC). Among CHB infection, immune-active (IA) patients with abnormal alanine aminotransferase (ALT) levels had relatively higher miR-155 expression in PBMCs and serum than immune-tolerant carriers, but were comparable to inactive carriers. Moreover, there was a positive correlation between miR-155 expression and ALT levels in CHB patients. Particularly, miR-155 expression in natural killer (NK) cells was significantly downregulated in IA patients compared with HC. Inversely, suppressor of cytokine signaling 1 (SOCS1), a target of miR-155, was upregulated in NK cells of IA patients. Overexpression of miR-155 in NK cells from IA patients led to a decrease in SOCS1 expression and an increase of IFN-γ production. Finally, accompanied by the normalization of ALT, miR-155 expression in PBMCs gradually decreased during telbivudine or peg-IFN-α-2a therapy. Interestingly, higher miR-155 expression at baseline was associated with better response to telbivudine therapy, but not peg-IFN-α-2a. In conclusion, our data suggested that miR-155 downregulation in NK cells of IA patients impaired IFN-γ production by targeting SOCS1, which may contribute to immune dysfunction during CHB infection. Additionally, baseline miR-155 expression could predict the treatment response to telbivudine therapy.

## Introduction

Hepatitis B virus (HBV) infection causes acute and chronic hepatitis and is a threat to public health across the world. It is estimated that 240 million people suffer from chronic HBV infections and are at risk of developing progressive liver diseases, such as cirrhosis, liver failure, and hepatocellular carcinoma (1). Generally, the outcome and pathogenesis of HBV infection is related to the complex interaction between viral activity and host immunity (2). MicroRNAs (miRNAs) are non-coding RNAs that regulate the expression of multiple genes at the posttranscriptional level by either translational repression or messenger RNA degradation (3). They play important roles in normal biological processes and have potential as disease biomarkers because they can indicate abnormal functions of particular organs. Many studies have reported that viral infections change host miRNAs profiles, which may affect virus–host interactions and participate in the viral life cycle and pathogenesis (4, 5). Currently, the available evidence indicates that miRNAs are involved in both the HBV life cycle and the development of HBV-associated liver diseases (6).

miR-155 is an evolutionarily conserved miRNA encoded in B-cell Integration Cluster non-coding RNA and acts as a crucial player in hematopoiesis, immune response, and inflammation (7). As miR-155 transcription is regulated by the activator protein-1 complex and the nuclear factor-κB transcription complex, the upregulation of miR-155 is often associated with increased cytokine release during the inflammatory process, particularly toll-like receptors (TLRs) signaling pathway activation (8, 9). Generally, miR-155 is involved in protective immunity when properly regulated and functions within a variety of activated immune cell types, including monocytes, natural killer (NK) cells, T cells, B cells, and dendritic cells (10). Within immune cells, miR-155 targets and represses many immune-regulatory proteins that include signaling molecules such as SH2-domain containing inositol-5-phosphatase 1 (SHIP1) (11) and suppressor of cytokine signaling 1 (SOCS1) (12), to regulate cytokines, chemokines, and transcription factors important for mounting an optimal immune response.

Because miR-155 also targets many important signaling proteins and transcription factors that govern immune processes and differentiation, it is not surprising that miR-155 has an important role during immune responses to HBV infection. Recently, Su et al. demonstrated that miR-155 could enhance innate antiviral immunity through promoting JAK/STAT signaling pathways by targeting SOCS1, and mildly inhibiting HBV replication in human hepatoma cells (13). Sarkar et al. found a positive correlation between TLR7 and miR-155 expression in HBV-infected liver biopsy and serum specimens as well as *in vitro*, which in turn modulated HBV replication (14). Yu et al. reported that expression of miR-155 was downregulated in peripheral blood mononuclear cells (PBMCs) and might correlate with the immune states of chronic hepatitis B (CHB) patients (15). However, the expression and role of miR-155 in regulating immune function during chronic HBV infection have yet to be explored.

Therefore, in the current study, we investigated the expression and function of miR-155 using (PBMCs) from CHB patients and its confirmatory expression in serum samples, as well as its alterations in PBMCs during therapy with telbivudine or pegylated interferon α-2a (peg-IFN-α-2a). The results provided new insights into the host antiviral response and the relationship between miR-155 expression and clinical outcome in patients with CHB.

## Materials and Methods

### Study Subjects

In the cross-sectional study, venous blood was drawn from 73 consecutive untreated CHB patients and 21 healthy controls (HC) at Nanfang Hospital (Guangzhou, China). According to the guidelines of the European Association for the Study of Liver Diseases (16), these CHB patients were divided into three groups of study subjects: immune-tolerant (IT) carriers (IT, *n* = 24), immune activation (IA, *n* = 27), and inactive carriers (IC, *n* = 22). The characteristics of all participants, with respect to demographic, biochemical, and virological features, are listed in Table 1.

**Table 1:** Clinical characteristics of the subjects for cross-sectional study.

Group	HC	IT	IA	IC
*n*	21	24	27	22
Gender (M/F)	10/11	13/11	20/7	10/12
Age (years)	25 (22–28)	27.5 (18–38)	28 (18–38)	28 (21–45)
HBV-DNA (log_10_ copies/ml)	ND	8.01 (6.72–8.82)	8.59 (6.37–9.48)	<3
ALT (U/l)	15 (6–38)	23.8 (14–41)	113 (55–593)	16.5 (10–39)
HBeAg/anti-HBe	0/0	24/0	27/0	0/22

*Values are *n* or median (range)*.

*M/F, male or female; ND, not determined; ALT, alanine aminotransferase; HBeAg, hepatitis B e antigen; HBV, hepatitis B virus; HC, healthy control; IT, immune tolerance; IA, immune activation; IC, inactive carrier*.

Forty-one HBeAg-positive CHB subjects who had participated in a prospective clinical trial of telbivudine (600 mg/day, clinical trial number: NCT00962533) (17) and another 24 patients from a prospective clinical trial of Peg-IFN-α-2a (180 μg/week, clinical trial number: NCT01086085) (18) in Nanfang Hospital were also studied.

All patients provided written documentation of informed consent to enter the study. The study protocol conformed to the ethical guidelines of the 1975 Declaration of Helsinki and was approved by the Ethical Committee of Nanfang Hospital.

### Serological Assays and HBV-DNA Assays

Serum HBsAg, HBeAg, anti-HBs, anti-HBe, and anti-HBc levels were quantitatively analyzed by the ARCHITECT i2000SR system (Abbott Ireland Diagnostics Division, Sligo, Ireland). Quantification of serum HBV-DNA was assayed by the COBAS TaqMan HBV Test (Roche Molecular Diagnostics, Pleasanton, CA, USA), which has a detection limit of 12 IU/ml or 69.84 copies/ml.

### PBMCs Isolation and Cell Subsets Sorting

Peripheral blood mononuclear cells were separated on Ficoll-Histopaque (BD Biosciences, Shanghai, China) density gradients and routinely cryopreserved as previously described (19). Thawed PBMCs were stained with anti-α-CD14-APC, anti-α-CD19-PE-Cy7, anti-α-CD3-APC-Cy7, and anti-α-CD56-FITC antibody (BD Biosciences, San Jose, CA, USA) for phenotype staining, while staining with 7AAD-PerCp (BD Biosciences) excluded dead cells. The stained cells were used for cell subset sorting on a BD FACSAria III (BD Biosciences). CD56^+^ NK cells were sorted from fresh PBMCs isolated from HCs (*n* = 12) and CHB patients (*n* = 12) with CD56 MicroBeads and MACS separation columns (Miltenyi Biotech, Shanghai, China), according to the manufacturer’s protocol. This purification protocol resulted in >95% purity of the selected cells, as determined by flow cytometry analysis (FACS) using anti-α-CD3-APC-Cy7 and anti-α-CD56-FITC antibody.

### RNA Extraction, Reverse Transcription, and Quantitative Real-time PCR

Total cellular RNA was isolated and purified from PBMCs or cell subsets using the miRNeasy miRNA isolation kit (Qiagen, Hilden, Germany) according to the manufacturer’s instructions. The cDNA synthesis was performed with 100 ng total miRNA using the miScript Reverse Transcription Kit (Qiagen). The expression of miR-155 genes was determined by real-time RT-PCR, which was performed with miScript SYBR Green PCR Kit (Qiagen) by using commercially available qPCR primer (Qiagen) on a LightCycler 480 (Roche Diagnostics International, Rotkreuz, Switzerland) according to the manufacturer’s instructions. The expression levels of each gene in the PBMCs were presented as values normalized against 10^6^ copies of U6 small nuclear RNA (RNU6B) transcripts. The miRNA in serum was extracted by miRNeasy Serum/Plasma Kits (Qiagen) and was synthesized as above. In addition, *C. elegans* miR-39 was added to serum samples as an internal control according to manufacturer’s recommends. Then quantitative real-time RT-PCR analysis for each gene in serum was performed as it was for the PBMCs, and the results were presented as values normalized against 10^6^ copies of *C. elegans* miR-39 miRNA transcripts. It was noteworthy that only 48 patients had enough corresponding serum retained to accomplish serum miR-155 detection.

### Cells Surface Staining and Flow Cytometry Analysis

Natural killer cells among the PBMCs were identified by flow cytometry analysis as the CD3^−^/CD56^+^ specimens. Expression of several activating (NKG2D, NKp46) and inhibitory (NKG2A, KIR3DL1/s1, Tim3) NK receptors, as well as secondary signals required for IFN-γ production (CD16) and early activation molecules (CD69) were examined by labeling with monoclonal antibody as described above.

### Cell Culture, Stimulation, and Functional Analysis

Fresh isolated NK cells were cultured at a density of 5 × 10^5^ cells/ml in RPMI 1640 (Life Technologies-Thermo Fisher Scientific Corp, Waltham, MA, USA) supplemented with 10% fetal bovine serum (FBS, Gibco, Grand Island, New York, NY, USA), 2 mM l-glutamine (Invitrogen, Carlsbad, CA, USA) and antibiotics/penicillin–streptomycin (Life Technologies, Carlsbad, CA, USA). NK cells were stimulated with IL-12 (10 ng/ml), IL-15 (10 ng/ml), and IL-18 (100 ng/ml) for 24 h as previously described (20). After 18 h of stimulation, 10 µg/ml of brefeldin A (BD Biosciences) was added to each well and incubated for the remaining 6 h for intracellular staining, while anti-α-CD107a-PE was added at the same time. Then the cultured cells were stained with LIVE/DEAD Fixable Near-IR Dead Cell Stain kit (life Technologies) to exclude dead cells and then the remaining viable cells were exposed to anti-α-CD3-PerCp, anti-α-CD56-APC, anti-α-CD3-PE-Cy7, anti-α-CD56-PerCp, anti-α-CD16-FITC, and anti-α-IFN-γ-PE, anti-α-TNF-α-FITC anti-α-Granzyme A-PE, anti-α-Perforin-APC, anti-α-Granzyme B-APC, or isotype antibody (BD Biosciences) for phenotype or intracellular staining, while with Fix/Perm A/B (Invitrogen) fixed and broken membranes. The stained cells were analyzed on a BD Canto II flow cytometer (BD Biosciences, San Jose, CA, USA).

### Transfection of NK Cells with miRNA Mimics

The hsa-miR-155-5p micrON™ miRNA mimic (miR-155 mimic) and miRNA mimic Ncontrol (miR-control) were synthesized by RiboBio (Guangzhou, China). CD56^+^ NK cells were sorted from fresh PBMCs isolated from IA patients (*n* = 6) with CD56 MicroBeads and MACS separation columns as described above. Then 5 × 10^5^ purified NK cells were resuspended in 0.5 ml of Gene Pulser electroporation buffer (Bio-Rad, CA, USA) with 100 nM miR-155 mimic or a miR-control, and were then transferred into Gene Pulser micropulser electroporation cuvettes (Bio-Rad). Transient transfection of the resuspended cells was performed using the Gene Pulser Xcell electroporation system (Bio-Rad), according to the manufacturer’s protocol. The transfected NK cells were immediately rescued after transfection in pre-warmed complete RPMI 1640 medium in 48-well culture plates, then incubated at 37°C and 5% CO_2_ for 3 days before analysis. Then the cells were stimulated and stained as above.

### Cytotoxic Activity of NK Cells

The separated NK cells (effector, E) were seeded onto 96-well plates at ratios of 5:1 with 1 × 10^4^ K562 (target cells-1, T-1), or at ratios of 2:1 with 1 × 10^4^ HepG2.2.15 (T-2), and then incubated with IL-12 (10 ng/ml), IL-15 (10 ng/ml), and IL-18 (100 ng/ml) for 18 h. The cytolytic activity of the NK cells was determined using a Cytotoxicity LDH Assay kit-WST (Dojindo Molecular Technologies, Japan) according to the manufacturer’s instructions.

### Statistical Analysis

Continuous data were shown as medians (minimum-maximum). Two-group comparisons were evaluated by the Mann–Whitney *U-*test, Wilcoxon signed-rank test, Chi-squared test, and paired *t-*tests. The Kruskal–Wallis *H* test was used when comparing more than three groups. Repeated measures analysis was used to compare changes in miR-155 expression during treatment. Differences in continuous variables were evaluated by paired Student’s tests. The correlations between variables were analyzed using the Spearman’s rank order correlation coefficient. Receiver-operating characteristic (ROC) curves were constructed to predict a CR to telbivudine treatment. All of the tests were two-tailed and a *p-*value of <0.05 calculated by SPSS Statistics 21.0 and GraphPad Prism 5.0 was considered statistically significant.

## Results

### miR-155 Expression Was Downregulated in PBMCs with Chronic HBV Infection and It Was Positively Correlated with ALT and TLR2 Levels

**Figure 1:**
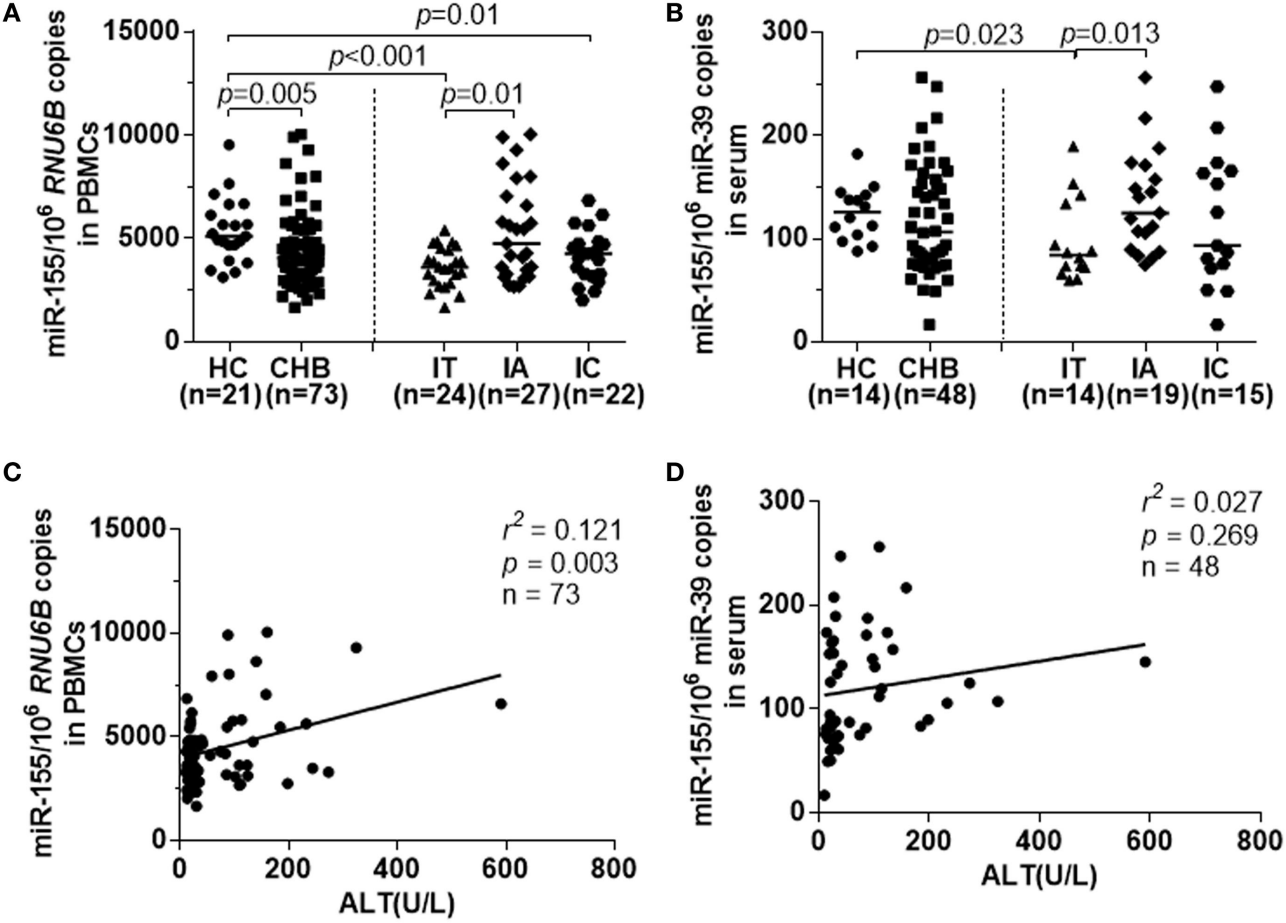
miR-155 expression in peripheral blood mononuclear cells (PBMCs) and serum from chronically hepatitis B virus (HBV)-infected patients and HCs. miR-155 expression were examined by real-time RT-PCR. **(A)** Comparison of miR-155 mRNA levels in PBMCs among healthy controls (HC), CHB, IT, IA, and IC groups. **(B)** Comparison of serum miR-155 mRNA levels among HC, CHB, IT, IA, and IC groups. **(C)** Spearman’s correlation of alanine aminotransferase (ALT) levels and miR-155 expression in PBMCs from all chronically HBV-infected patients. **(D)** Spearman’s correlation of ALT levels and miR-155 expression in serum from all chronically HBV-infected patients. HC, healthy control; CHB, chronic hepatitis B; IT, immune tolerance; IA, immune activation; IC, inactive carrier.

For the first step, we examined the expression of miR-155 in PBMCs and serum samples from all CHB patients by real-time RT-PCR. The miR-155 levels were lower in PBMCs from CHB patients than from HCs (Figure 1A, *p* = 0.005). The median miR-155 levels in PBMCs were downregulated in both the IT and IC groups compared to the HC group (Figure 1A, HC vs IT, *p* < 0.001; HC vs IC, *p* = 0.01). Interestingly, the miR-155 expression was higher in IA patients with abnormal ALT levels (Figure 1A, IA vs IT, *p* = 0.01). At the same time, miR-155 serum levels during various phases of chronic HBV infection were similar to those occurring in PBMCs (Figure 1B, HC vs IT, *p* = 0.023; IA vs IT, *p* = 0.013). Although serum miR-155 expression in CHB patients had a tendency to be lower than in the HC group, these differences were not statistically significant (Figure 1B). No correlation was found between miR-155 expression in PBMCs and serum miR-155 levels (Figure S1A in Supplementary Material).

We then evaluated the association between ALT levels, HBV DNA, and miR-155 mRNA expression in PBMCs and serum of the subjects with chronic HBV infections. As shown in Figure 1C, Spearman analysis revealed that there was a positive correlation between miR-155 levels in PBMCs and ALT levels (*r^2^* = 0.121, *p* = 0.003), but no correlation was observed between miR-155 levels in PBMCs and HBV DNA loads (Figure S1B in Supplementary Material, *p* = 0.281). However, there was no correlation between miR-155 levels in serum and ALT (Figure 1D, *p* = 0.269). As TLRs had been reported to mediate miR-155 expression and function during HBV infection (14), we also examined the relationship between miR-155 and TLR2 and found there was a high positive correlation between them (Figure S1C in Supplementary Material, *r^2^* = 0.305, *p* < 0.001).

In contrast to miR-155, miR-146a is a negative modulator of innate and adaptive immunity and its upregulation in CHB causes impaired T cell function, which may contribute to immune defects and immunopathogenesis that develops during chronic HBV infection (21). We also examined the miR-146a expression in PBMCs and serum samples of CHB. Although miR-146a and miR-155 had a positive correlation in PBMCs (Figure S2A in Supplementary Material), there was no significant difference of miR-146a expression in PBMCs and serum from all CHB patients or patients with different phases of chronic HBV infection (Figures S2B,C in Supplementary Material).

### Reduced miR-155 Expression Was Associated with Dysfunction of NK Cells in CHB Patients

**Figure 2:**
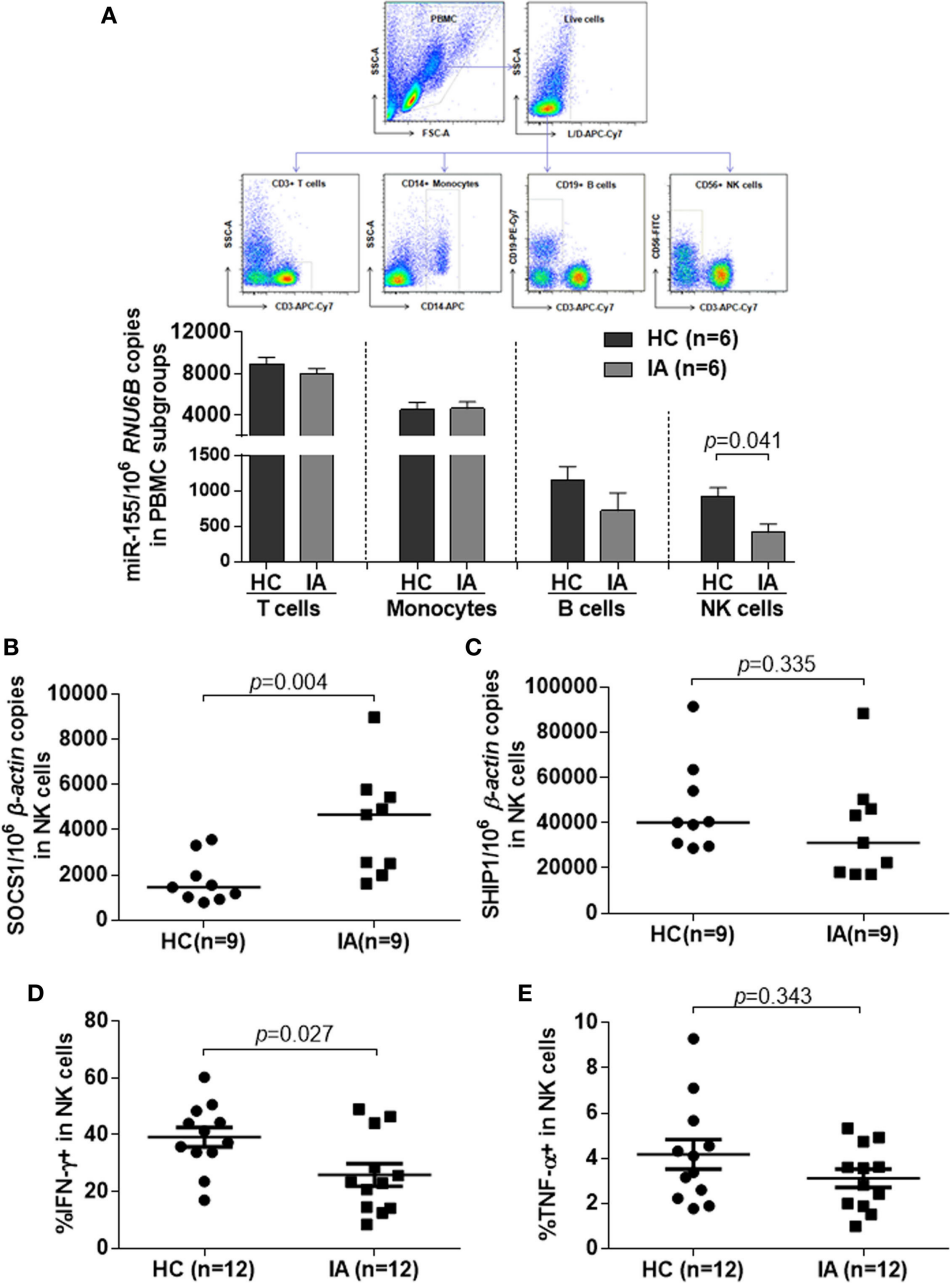
miR-155 mRNA levels and cytokines secretion in natural killer (NK) cells was significantly downregulated in IA patients compared with HCs, while suppressor of cytokine signaling 1 (SOCS1) was upregulated in NK cells of IA group. **(A)** Purity of T, B, NK cells, and monocytes isolated from the healthy controls (HC) and IA groups by fluorescent cell sorting. miR-155 expression in different cell subsets was determined by real-time RT-PCR. **(B,C)** NK cells were isolated from peripheral blood mononuclear cells of HC and IA group by CD56 MicroBeads. SOCS1 **(B)** and SHIP1 **(C)** mRNA expression were determined by real-time RT-PCR. **(D,E)** NK cells were stimulated with IL-12 (10 ng/ml), IL-15 (10 ng/ml), and IL-18 (100 ng/ml) for 24 h. IFN-γ **(D)** and TNF-α **(E)** secretion were examined by flow cytometric analysis. HC, healthy control; IA, immune activation.

Chronic HBV infection is a complicated process, especially in the IA phase. Abnormal ALT levels generally reflect the inflammation activity accompanied with immune activation in these patients. According to EASL guideline, under normal circumstances, only IA patients, but not IT and IC, need to be treated. However, impaired immune function in IA patients could not eliminate HBV completely, which result in HBV persistence. To further determine the effect of immune dysfunction of IA patients on miR-155 expression, we measured miR-155 levels by real-time RT-PCR in subpopulation of PBMCs from HBV-infected subjects. Compared with non-infected HCs, although the miR-155 levels of IA patients were comparable in whole PBMCs, their expression still were significantly decreased in CD56^+^ NK cells (*p* = 0.041), but not in CD3^+^ T cells, CD14^+^ monocytes, or CD19^+^ B cells (Figure 2A). As mentioned above, SOCS1 and SHIP1 are two direct targets of miR-155, validated in many cell types. We next examined the expression of these two genes in NK cells from HC and IA. The mRNA level of SOCS1 was much higher in IA patients than in HCs (Figure 2B, *p* = 0.004), while the mRNA levels of SHIP1 were not significantly different (Figure 2C). SOCS1 and SHIP1 were reported to control IFN signaling and cytokine production in NK cells. Consistently, NK cells isolated from IA patients displayed decreased IFN-γ (Figure 2D, *p* = 0.027) and relatively ordinary TNF-α (Figure 2E, *p* = 0.343) secretion compared with NK cells from HCs after stimulation with IL-12, IL-15, and IL-18. These results suggested that reduced expression of miR-155 in NK cells might contribute to lower cytokine production in activated NK cells. As defects in activation and antiviral function of NK cells having been described in patients with chronic HBV infection (22, 23), we also determined several activating (NKG2D, NKp46) and inhibitory (NKG2A, KIR3DL1/s1, Tim3) NKRs, as well as secondary signal required for IFN-γ production (CD16), early activation molecules (CD69), and cytotoxicity factors (Perforin, CD107a, Granzyme A, Granzyme B) in NK cells between IA patients and HCs. We found that there were similar lower perforin secretion (Figures S3A,B in Supplementary Material, *p* = 0.009) and NKG2D expression (Figures S3A,F,G in Supplementary Material, %, *p* = 0.002, MFI, *p* = 0.001) of NK cells in IA patients than HCs, while the secretion of CD107a, Granzyme A, and Granzyme B (Figures S3A,C–E in Supplementary Material) and the expression of NKG2A, KIR3DL1/s1, Tim3, CD16, CD69 had no significant differences in IA patients and HCs (data not shown).

### Overexpression of miR-155 Led to Decrease of SOCS1 Expression and Increase of Cytokine Production in Activated NK Cells

**Figure 3:**
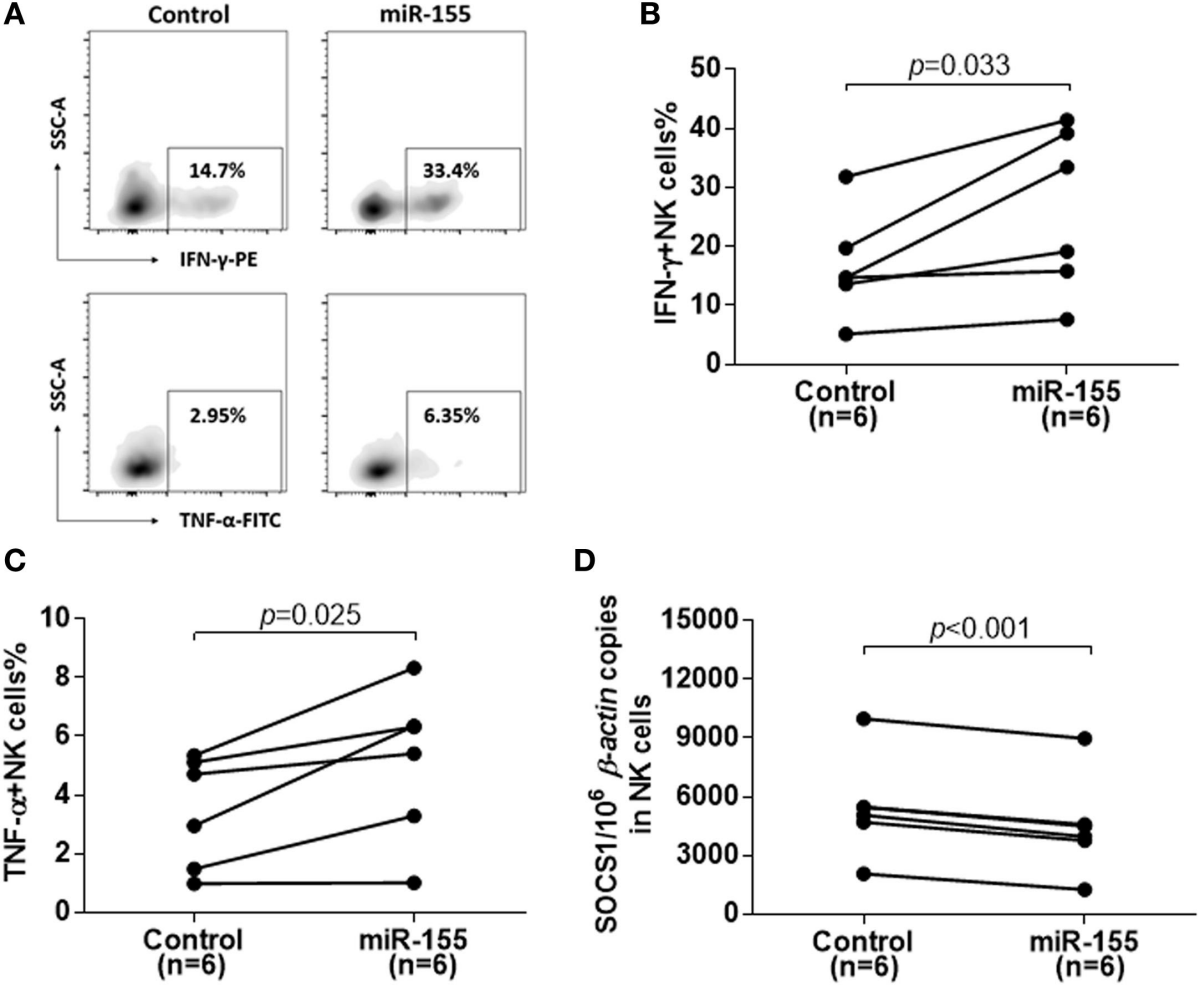
Reconstitution of miR-155 in natural killer (NK) cells from immune-activation (IA) patients led to decreases in suppressor of cytokine signaling 1 (SOCS1) expression and increases in cytokine production. Purity of NK cells from IA patients was transfection with miR-control or miR-155-mimic for 72 h. **(A–C)** After stimulated with IL-12 (10 ng/ml), IL-15 (10 ng/ml), and IL-18 (100 ng/ml) for 24 h, IFN-γ and TNF-α secretion of isolated NK cells were detected by flow cytometric analysis. A representative pattern **(A)** and statistics analysis of IFN-γ **(B)** and TNF-α **(C)** secretion of transfected NK cells determined by flow cytometric analysis. **(D)** After 72 h transfection, SOCS1 mRNA expression in isolated NK cells was determined by real-time RT-PCR. Control, miR-control; miR-155, miR-155 mimic.

To confirm the role of miR-155 in cytokine production in NK cells after activation, we examined the IFN-γ and TNF-α production after miR-155 overexpression in NK cells from IA patients. miR-155 mimics or miR-controls were transfected into NK cells by electronic transduction and about a threefold increase in the miR-155 level was detected by real-time RT-PCR analysis. Compared to miR-controls, electronic transduction of NK cells with the miR-155 mimics resulted in an increase of IFN-γ^+^ and TNF-α^+^ NK cells after stimulation with IL-12, IL-15, and IL-18 (Figure 3A). The frequency of IFN-γ^+^ (*p* = 0.033) and TNF-α^+^ (*p* = 0.025) NK cells were significantly higher in the miR-155 mimic group than in the miR-control group (Figures 3B,C). Moreover, miR-155 mimics transfected NK cells displayed a decrease in target gene SOCS1 expression (Figure 3D, *p* < 0.001). Meanwhile, to further analysis the effect of miR-155 in NK cells activity and cytotoxicity, we also determined the aforementioned factors. Regrettably, miR-155 upregulation would not increase the secretion of perforin (Figures S4A,C in Supplementary Material) and expression of NKG2D (Figures S4G–I in Supplementary Material) or other factors (Figures S4A,D–F in Supplementary Material) and expression of NKG2D, but only elevated the expression of CD69 after IL-12, IL-15, and IL-18 stimulation (Figures S4A,B in Supplementary Material). Consistent with the results above, overexpression of miR-155 did not promote the cytolytic activity of NK cells on K562 or HepG2.2.15 target cells (Figures S4J,K in Supplementary Material). These results suggested that miR-155 might act as a positive regulatory molecule for NK cell-mediated cytokine production, but might not have an effect on the cytotoxicity of NK cells.

### Higher Baseline miR-155 Levels Was Associated with the Treatment Response to Telbivudine Therapy

**Figure 4:**
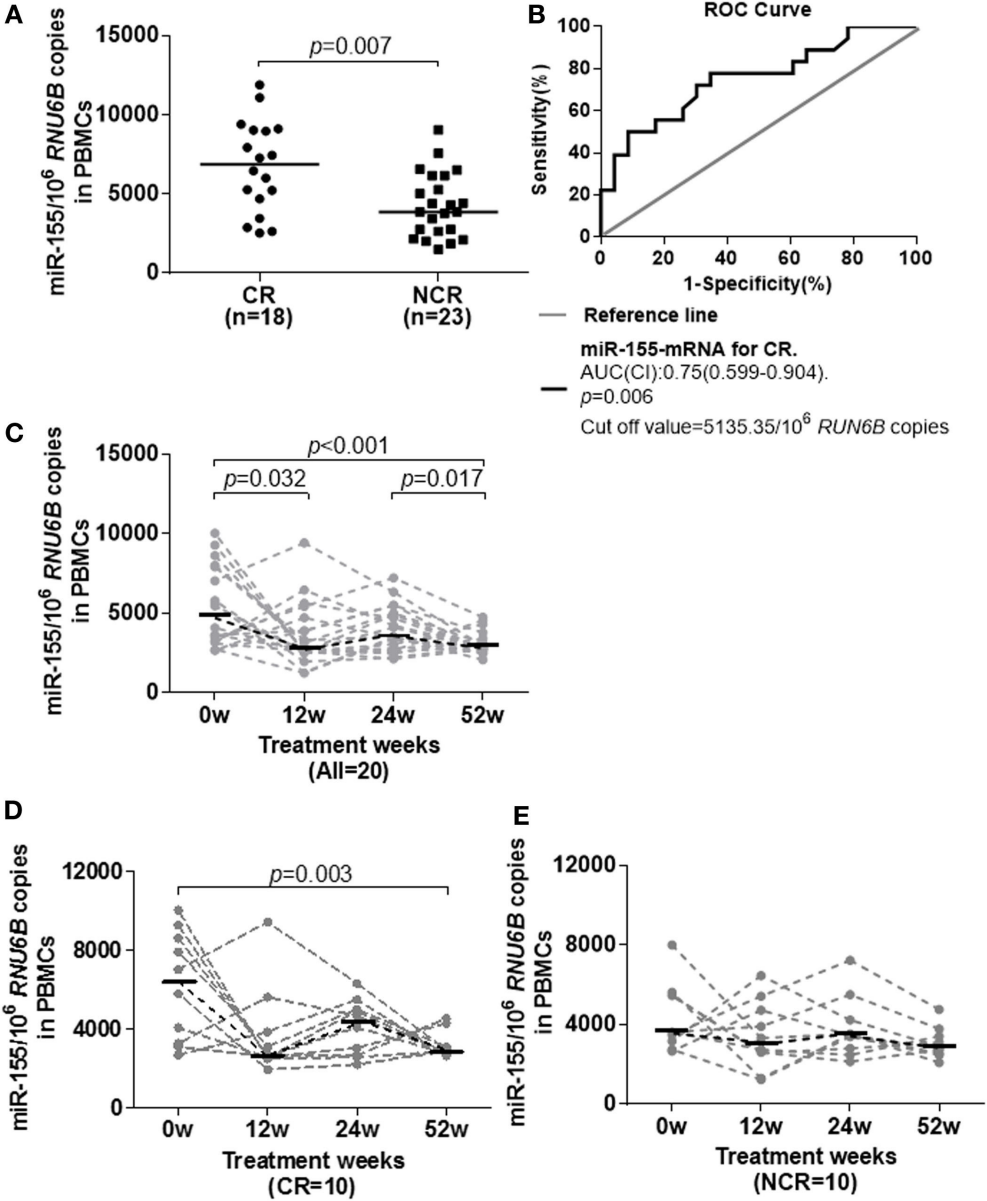
Longitudinal analysis of miR-155 expression in peripheral blood mononuclear cells (PBMCs) of IA patients during telbivudine therapy. **(A)** miR-155 mRNA levels in PBMCs at baseline in the CR and NCR groups. **(B)** Receiver-operating characteristic (ROC) curve showed the suitability of miR-155 mRNA levels at baseline to predict a CR to telbivudine therapy. An area under the curve (AUC) of 1.0 occurs with an ideal test, whereas an AUC of <0.5 indicates a test of no diagnostic value. **(C)** Temporal dynamics of miR-155 mRNA levels in PBMCs of all patients. **(D)** Comparison of miR-155 mRNA levels among individuals in the CR group. **(E)** Comparison of miR-155 mRNA levels among individuals in the NCR group. IA, immune activation; CR, complete response; NCR, non-complete response.

Considering that miR-155 may be beneficial for cytokine-mediated antiviral function, we investigated whether miR-155 expression was associated with the antiviral response to nucleoside analog therapy. The miR-155 levels in PBMCs were examined in 41 treatment naïve HBeAg-positive IA patients who received telbivudine treatment. They were divided into complete response (CR, *n* = 18) and non-complete response (NCR, *n* = 23) groups according to their treatment outcomes at week 52. Patients in the CR group had a normalized ALT level and HBeAg seroconversion and achieved a reduction of the serum HBV DNA level to <300 copies/ml at week 52, while those in the NCR group had either a serum HBV DNA level >300 copies/ml or were positive for HBeAg at week 52 (24). The baseline clinical features of CR and NCR patients were comparable, and on-treatment HBV-DNA, HBsAg, and HBeAg were significantly lower in CR than NCR at week 12, week 24, and week 52 (Table 2). Interestingly, miR-155 expression in PBMCs from the CR group at the initiation of treatment was significantly higher than in those from the NCR group (Figure 4A, *p* = 0.007). A ROC curve was generated to assess the usefulness of baseline miR-155 levels to predict a CR at week 52. The optimal cut-off value for the miR-155 expression in PBMCs was 5,135.35/10^6^ *RUN6B* copies. This indicated that sensitivity for detection of a CR was 72.2% with a specificity of 69.6% (Figure 4B). These results suggested that a higher level of miR-155 expression at baseline might be conducive to the immune control of HBV during nucleoside analog treatment. In the meantime, we also examined the expression of miR-146a in PBMCs between CR and NCR groups at baseline, unfortunately, there was no any differences between the two groups (Figure S5A in Supplementary Material).

**Table 2:** Clinical characteristics of the subjects under telbivudine therapy for longitudinal study.

Group		CR	NCR	*p-*Value
*N*		18	23	
Gender (M/F)		15/3	21/2	0.638^a^
Age (years)		27.5 (18–42)	28 (18–40)	0.989^b^
0 weeks	HBV-DNA (log_10_ copies/ml)	8.52 (5.78–9.43)	8.63 (6.56–9.7)	0.121^b^
	ALT (U/l)	118 (60–830)	130 (55–324)	0.655^b^
	HBsAg (log_10_ IU/ml)	4.16 (2.53–4.66)	4.45 (3.24–5.07)	0.088^b^
	HBeAg (log_10_ PEIU/ml)	2.38 (0.36–3.79)	3.11 (1.33–3.93)	0.189^b^

12 weeks	HBV-DNA (log_10_ copies/ml)	3.13 (2.08–4.97)	3.99 (2.34–5.93)	0.009^b^
	ALT (U/l)	29.5 (15–160)	40 (16–211)	0.098^b^
	HBsAg (log_10_ IU/ml)	ND	ND	
	HBeAg (P/N)	12/6	23/0	0.004^a^

24 weeks	HBV-DNA (log_10_ copies/ml)	2.38 (1.84–4.09)	3.01 (1.84–5.27)	0.025^b^
	ALT (U/l)	23.5 (14–65)	25 (14–63)	0.493^b^
	HBsAg (log_10_ IU/ml)	3.24 (2.07–4.21)	3.74 (3.09–4.69)	0.009^b^
	HBeAg (P/N)	11/7	23/0	0.001^a^

52 weeks	HBV-DNA (log_10_ copies/ml)	1.84 (1.84–2.09)	1.84 (1.84–4.840)	0.006^b^
	ALT (U/l)	19.5 (13–56)	19 (12–52)	0.544^b^
	HBsAg (log_10_ IU/ml)	3.34 (1.88–4.11)	3.86 (2.90–4.76)	0.006^b^
	HbeAg (P/N)	0/18	23/0	0.000^a^

*Values are *n* or median (range)*.

*^a^Chi-squared test*.

*^b^Mann–Whitney U-test*.

*M/F, male or female; ND, not determined; P/N, positive or negative; CR, complete response, patients with HBeAg seroconversion at week 52; NCR, non-complete response, patients without HBeAg seroconversion at week 52; ALT, alanine aminotransferase; HBeAg, hepatitis B e antigen; HBsAg, hepatitis B surface antigen; HBV, hepatitis B virus*.

We also examined the dynamic change of miR-155 expression during telbivudine therapy. Available PBMCs from 20 IA patients (including 10 CR and 10 NCR) were followed prospectively for analysis and exhibited a significant reduction of miR-155 expression at week 12 (*p* = 0.032) and week 52 (*p* = 0.017, Figure 4C). As analyzed separately, miR-155 expression in NCR patients showed a steady trend with no obvious decrease (Figure 4D). However, CR patients displayed reduced miR-155 expression but reached statistically significant differences only at week 52 (Figure 4E, *p* = 0.003). Moreover, we observed no significant difference of miR-146 expression after the start of therapy between the CR and NCR groups (data not shown).

### Gradual Decline of miR-155 Expression in PBMCs during Peg-IFN-α-2a Antiviral Therapy

**Figure 5:**
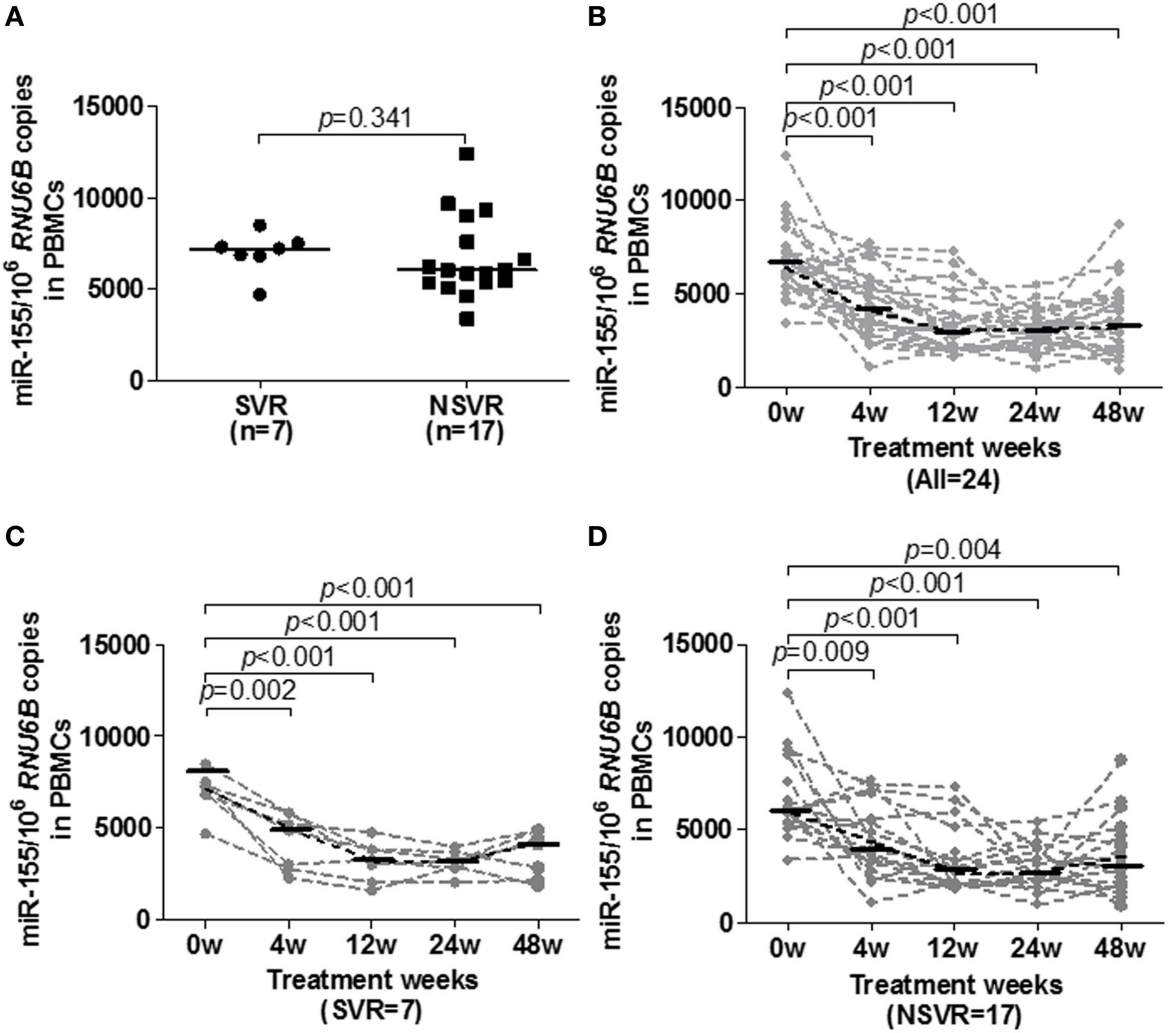
Longitudinal analysis of miR-155 expression in peripheral blood mononuclear cells (PBMCs) of IA patients during peg-IFN-α-2a therapy. **(A)** miR-155 mRNA levels in PBMCs at baseline in the SVR and NSVR groups. **(B)** Temporal dynamics of miR-155 mRNA levels in PBMCs of all patients. **(C)** Comparison of miR-155 mRNA levels among individuals in the SVR group. **(D)** Comparison of miR-155 mRNA levels among individuals in the NSVR group. IA, immune activation; SVR, off-treatment sustained virological response; NSVR, non-off-treatment sustained virological response.

Additionally, we tested the baseline and dynamic changes of miR-155 expression in PBMCs of IA patients during peg-IFN-α-2a therapy. A total of 24 treatment naïve IA patients who received 48 weeks of peg-IFN-α-2a treatment were divided into a sustained virological response (SVR, *n* = 7) group and a non-sustained virological response (NSVR, *n* = 17) group according to their treatment responses at week 72. Subjects in the SVR group had undergone HBeAg seroconversion and had achieved a serum HBV DNA levels <1,000 copies/ml, while NSVR group had either serum HBV DNA levels >1,000 copies/ml or were positive for HBeAg. The baseline clinical features of SVR and NSVR patients were comparable, and HBV DNA and HBsAg were significantly lower in the SVR group than the NSVR group at weeks 48 and 72 (Table 3). However, unlike in the telbivudine treatment groups, there were no significant differences of miR-155 expression at the baseline of therapy (Figure 5A, *p* = 0.3408). Moreover, all the patients exhibited gradually decreasing miR-155 expression during treatment (Figure 5B). By comparing the SVR and NSVR groups, we found that miR-155 expression in both decreased gradually after starting treatment and showed no significant differences during the course of treatment (Figures 5C,D). Meanwhile, we also examined the expression of miR-146a in PBMCs between SVR and NSVR groups at baseline, but there was still no any differences between the two groups (Figure S5B in Supplementary Material), too.

**Table 3:** Clinical characteristics of the subjects under peg-IFN-α-2a therapy for longitudinal study.

	Group	SVR	NSVR	*p-*Value
*n*		7	17	
Gender (M/F)		6/1	13/4	1.000^a^
Age (years)		25 (21–34)	28 (21–43)	0.349^b^
0 weeks	HBV-DNA (log_10_ copies/ml)	8.37 (5.83–9.72)	8.49 (6.00–9.94)	0.576^b^
	ALT (U/l)	249 (44–384)	172 (46–400)	0.349^b^
	HBsAg (log_10_ IU/ml)	3.38 (1.23–5.10)	4.12 (2.83–5.10)	0.166^b^
	HBeAg (log_10_ PEIU/ml)	2.52 (0.41–3.68)	3.10 (−0.52–4.01)	0.664^b^

4 weeks	HBV-DNA (log_10_ copies/ml)	6.47 (3.56–8.05)	7.50 (4.29–9.56)	0.288^b^
	ALT (U/l)	137 (55–384)	121 (22–275)	0.383^b^
	HBsAg (log_10_ IU/ml)	3.73 (1.46–4.51)	3.62 (2.18–4.92)	0.664^b^
	HBeAg (P/N)	ND	ND	

12 weeks	HBV-DNA (log_10_ copies/ml)	5.65 (2.54–6.79)	6.00 (3.31–8.80)	0.664^b^
	ALT (U/l)	84 (28–136)	46 (16–203)	0.418^b^
	HBsAg (log_10_ IU/ml)	2.97 (1.68–3.90)	3.37 (2.17–4.48)	0.099^b^
	HBeAg (P/N)	6/1	16/1	0.507^a^

24 weeks	HBV-DNA (log_10_ copies/ml)	4.77 (2.46–5.84)	4.78 (2.46–9.49)	0.383^b^
	ALT (U/l)	41 (25–85)	41 (18–129)	0.852^b^
	HBsAg (log_10_ IU/ml)	3.05 (1.64–3.86)	3.36 (1.60–4.53)	0.349^b^
	HBeAg (P/N)	5/2	16/1	0.194^a^

48 weeks	HBV-DNA (log_10_ copies/ml)	2.46 (2.46–4.70)	4.34 (2.46–8.42)	0.016^b^
	ALT (U/l)	41 (19–62)	35 (4.05–100)	0.534^b^
	HBsAg (log_10_ IU/ml)	1.79 (1.12–3.90)	3.21 (1.15–4.33)	0.047^b^
	HBeAg (P/N)	¾	15/2	0.038^a^

72 weeks	HBV-DNA (log_10_ copies/ml)	2.46 (2.46–2.79)	4.93 (2.46–9.68)	0.000^b^
	ALT (U/l)	20 (8–47)	22 (8–351)	0.494^b^
	HBsAg (log_10_ IU/ml)	2.37 (−0.33–3.85)	3.26 (1.04–5.10)	0.019^b^
	HBeAg (P/N)	0/7	15/2	0.000^a^

*Values are *n* or median (range)*.

*^a^Chi-squared test*.

*^b^Mann–Whitney U-test*.

*M/F, male or female; ND, not determined; P/N, positive or negative; SVR, off-treatment sustained virological response; NSVR, non- off-treatment sustained virological response; ALT, alanine aminotransferase; HBeAg, hepatitis B e antigen; HBsAg, hepatitis B surface antigen; HBV, hepatitis B virus*.

## Discussion

In the present study, we investigated the relevance of miR-155 for impaired NK cell function during chronic HBV infection. We found that miR-155 levels were significantly decreased in both PBMCs and serum of our subjects with chronic HBV infection, especially in the subjects who were IT or IC. However, miR-155 expression was comparable to HCs in the IA phase of CHB, which may result from chronic inflammation. Further analysis showed that lower expression of miR-155 was accompanied by a suppression of cytokine production of NK cells in IA patients. Finally, higher miR-155 expression was associated with a complete response to telbivudine therapy and a reduction of miR-155 was observed during both telbivudine and peg-IFN-α-2a therapy.

As a key modulator of the immune response and inflammation, miR-155 is dysregulated in various infectious diseases. For example, Yang et al. demonstrated the role of the TLR2/miR-155/SOCS1 signaling axis in the immunity and apoptosis of macrophages during the innate immune response to mycobacterial infections (25). Sarkar et al. reported that miR-155 was suppressed during HBV infection and a subsequent positive correlation of miR-155 with TLR7 activation (14). These findings led us to further explore the relationship between miR-155 and TLRs. We found that TLR2 had a positive correlation with miR-155 expression in PBMCs from CHB patients. Consistently, there was a positive correlation between miR-155 and liver inflammation-related abnormal ALT levels, but no correlation was observed between miR-155 and HBV DNA loads. These findings suggested that HBV infection might not influence the miR-155 expression directly, but affect the expression by mediating immune responses and inducing inflammatory cytokines (13). Unexpectedly, although miR-155 level in serum exhibited a similar expression to that in PBMCs, there was no correlation between miR-155 expression in PBMCs and serum, in parallel to the lack of relationship between miR-155 levels in serum and the ALT level. It is possible that, *in vivo*, miR-155 is ubiquitously expressed, not only in many immune cell types but also in human reproductive tissues, fibroblasts, epithelial tissues, and the central nervous system (26).

Moreover, our findings revealed that downregulation of miR-155 in NK cells resulted in the dysfunction of these cells in IA patients. Importantly, overexpression of miR-155 could lead to a decrease of SOCS1 expression and an increase of cytokine production in activated NK cells. SOCS1 was involved in negatively regulating JAK–STAT signaling (27) and mice lacking SOCS1 had increased levels of IFN-γ in the serum (28). It was also shown that endogenous SOCS1 participate in the prevention of liver diseases such as hepatitis, fibrosis, and cancers (29, 30). Recently, the H3K4me3 demethylase Kdm5a was found to be required for priming activation of NK cells by suppressing the SOCS1 epigenetically (31). NK cells play an essential role in liver immunity and act as the first line of defense against invading pathogens. They generally become activated and eliminate HBV-infected cells directly by cytolytic killing and indirectly by secreting cytokines (32, 33). Nevertheless, the frequency, activation, and cytokine production by circulating NK cells were significantly reduced in patients with HBV infection (34). Consistent with a previous study (35), our study also documented that NK cells from IA patients produce less IFN-γ and TNF-α during stimulation with IL-12, IL-15 and IL-18, compared to that which occurs in HCs. Reconstitution of miR-155 in NK cells could lead to an increase in IFN-γ and TNF-α production. Since previous studies indicated that SHIP1 (36) and SOCS1 (37) are direct targets of miR-155, and are involved in control of cytokine production and the cytolytic function of NK cells (38). We found significantly higher expression of SOCS1 in NK cells from the IA group than in those from the HC group. Furthermore, SOCS1 expression was decreased upon overexpression of miR-155 in NK cells, which has an inverse trend with antiviral cytokine secretion. Together with two previous studies showing that ectopic expression of miR-155 in hepatoma cells mildly inhibited HBV infection by suppressing SOCS1 and subsequently upregulating the expression of several IFN-inducible antiviral genes (13, 14), our current work suggests that miR-155 might act as a positive regulator in miR-155/SOCS1/IFN-γ negative feedback loop of the innate immune system during chronic HBV infection. Regrettably, the upregulation of miR-155 seem to have no effect on NK cytotoxicity. There might be some other regulators in NK cell for its cytolytic activity.

Besides natural HBV infection, host immune status is regulated and associated with the treatment outcome of antiviral therapy (39). In both telbivudine and peg-IFN-α-2a-treated patients, there was a steady decline of miR-155 level during antiviral therapy. It was likely that the decrease and normalization of ALT level by antiviral treatment reduced the inflammation, resulted in the continuous reduction of miR-155 expression. This trend was consistent with the expression of TLR2 and TLR8 during antiviral treatment as our previous findings (24, 40). One of the important goals in the treatment of IA patients is to achieve HBeAg seroconversion, which is closely related to sustained inhibition of HBV DNA levels (41). A previous report had revealed that the profile of plasma miRNAs may predict an early virological response to IFN treatment in IA patients (42). Furthermore, miR-155 has been found to negatively correlate with viral load in patients with HCV infection and might be associated with an efficient antiviral response against HCV (43). In this study, we noted that the baseline miR-155 level in PBMCs was correlated with the treatment response to telbivudine, but not peg-IFN-α-2a. The intrinsic mechanisms for this discrepancy might be associated with the interaction of multiple immune factors that contribute to viral pathogenesis or the different antiviral mechanisms of these two drugs (39).

In conclusion, our study indicates that chronic HBV infection may inhibit miR-155 expression and produce miR-155 dysregulation in NK cells. This may subsequently lead to suppression of NK function by targeting SOCS1. Additionally, we established a positive correlation between miR-155 expression and treatment outcomes of antiviral therapy. Given the broad function of miR-155 in both the innate and adaptive immune responses, our work provides valuable insight for functional implications of miR-155 in HBV infection, implying miR-155 may be a positive regulator contributing to the functional impairment of NK cells and viral persistence during chronic HBV infection. Understanding how miR-155 functions in the complex regulation networks involved in the immune-pathogenesis of chronic HBV infection will help us to identify novel therapeutic targets for treatment of CHB.

## Ethics Statement

All patients provided written documentation of informed consent. The study conformed to the ethical guidelines of the 1975 Declaration of Helsinki and was approved by the Ethical Committee of Nanfang Hospital.

## Author Contributions

XZ conceived and designed the study. JG, ZH, HL, JC, ZX, and ZC performed the experiments and analyzed the data. XZ and JG wrote the manuscript with additional input and suggestions from JP, JS, and JH. All authors reviewed and approved the manuscript.

## Conflict of Interest Statement

The authors declare that the research was conducted in the absence of any commercial or financial relationships that could be construed as a potential conflict of interest.

## Abbreviations

ALT, alanine aminotransferase; CHB, chronic hepatitis B; CR, complete response; HBeAg, hepatitis B e antigen; HBsAg, hepatitis B surface antigen; HBV, hepatitis B virus; HC, healthy control; IA, immune activation; IC, inactive carrier; IFN, interferon; IL, interleukin; IT, immune tolerance; miRNA, microRNA; NCR, non-complete response; NK cell, nature killer cell; NSVR, non-sustained virological response; PBMC, peripheral blood mononuclear cell; peg-IFN-α-2a, pegylated interferon α-2a; SHIP1, SH2-domain containing inositol-5-phosphatase 1; SOCS1, suppressor of cytokine signaling 1; SVR, sustained virological response; TLR, toll-like receptor; TNF-α, tumor necrosis factor α.
