# Supplementary material for: Lower Expression of MicroRNA-155 Contributes to Dysfunction of Natural Killer Cells in Patients with Chronic Hepatitis B
Source: Front Immunol. 2017 Sep 22;8:1173. doi: 10.3389/fimmu.2017.01173 (PMC5614978; doi:10.3389/fimmu.2017.01173)
Supplement: Supplementary file 1 [file data_sheet_1.docx]

**SupplementAL Figure Legends**

**Supplemental Figure 1:** The correlations of miR-155 expression in PBMCs with which in serum or other indicators in CHB patients. (A) Spearman's correlation of miR-155 mRNA levels in PBMCs and serum from CHB patients. (B) Spearman's correlation of HBV DNA levels and miR-155 expression in PBMCs from CHB patients. (C) Spearman's correlation of TLR2 mRNA and miR-155 expression in PBMCs from CHB patients. CHB, chronic hepatitis B.

**Supplemental Figure 2:** miR-146 expression in PBMCs and serum from chronic CHB patients and HCs. (A) Spearman's correlation of miR-155 and miR-146 expression in PBMCs from CHB patients. (B) Comparison of miR-146 mRNA levels in PBMCs among HC, CHB, IT, IA and IC groups. (C) Comparison of serum miR-146 mRNA levels among HC, CHB, IT, IA and IC groups. HC, healthy control; CHB, chronic hepatitis B; IT: immune tolerance; IA, immune activation; IC, inactive carrier.

**Supplemental Figure 3:** The surface markers expressions and cytolytic factors secretions of NK cells between IA patients and HCs. NK cells were isolated from PBMCs of HC and IA group by CD56 MicroBeads. Purified NK cells from IA patients and HCs was detected the expression of surface markers immediately, or stimulated with IL-12 (10 ng/ml), IL-15 (10 ng/ml) and IL- 18 (100 ng/ml) for 24 h to exam the secretions of cytolytic factors by flow cytometric analysis. A representative pattern (A) and statistics analysis of perforin (B), CD107a (C), Granzyme A (D) and Granzyme B (E) secretions of NK cells determined after 24 h stimulation. NKG2A expressions (F) and its MFI values of NK cells (G) were determined by flow cytometric analysis. HC, healthy control; IA, immune activation; MFI, mean fluorescence intensity.

**Supplemental Figure 4:** Reconstitution of miR-155 in NK cells from IA patients led to increased CD69 expression, but not significant variation of cytolytic acivity. Purity of NK cells from IA patients was transfection with miR-control or miR-155-mimic for 72 h. (A-I) The expressions of surface markers were detected by flow cytometric analysis immediately, while the secretions of cytolytic factors were examined after 24 h IL-12 (10 ng/ml), IL-15 (10 ng/ml) and IL- 18 (100 ng/ml) stimulation. A representative pattern (A) and statistics analysis of CD69 expression (B) and perforin (C), CD107a (D), Granzyme A (E) and Granzyme B (F) secretions of transfected NK cells after 24h stimulation were determined by flow cytometric analysis. A representative pattern (G) and statistics analysis of NKG2A expression (H) and its MFI values (I) were determined immediately. (J) The cytotoxicity of K562 by transected NK cells after 18 h IL-12 (10 ng/ml), IL-15 (10 ng/ml) and IL- 18 (100 ng/ml) stimulation were analyzed by LDH assay. (J) The cytotoxicity of HepG2.2.15 by transfected NK cells after 18 h of IL-12 (10 ng/ml), IL-15 (10 ng/ml) and IL- 18 (100 ng/ml) stimulation were analyzed by LDH assay. Control, miR-control; miR-155, miR-155 mimic; IA, immune activation; MFI, mean fluorescence intensity.

**Supplemental Figure 5:** Correlation of miR-146 expression in PBMCs at baseline with treatment response of IA patients during antiviral therapy. (A) miR-146 mRNA levels in PBMCs at baseline in the CR and NCR groups with telbivudine therapy. (B) miR-146 mRNA levels in PBMCs at baseline in the SVR and NSVR groups with peg-IFN-α-2a therapy. IA, immune activation; CR, complete response; NCR, non-complete response; SVR, off-treatment sustained virological response; NSVR, non-off-treatment sustained virological response.
