# Supplementary figures and images for: Lower Expression of MicroRNA-155 Contributes to Dysfunction of Natural Killer Cells in Patients with Chronic Hepatitis B
Source: Front Immunol. 2017 Sep 22;8:1173. doi: 10.3389/fimmu.2017.01173 (PMC5614978; doi:10.3389/fimmu.2017.01173)

## Suppl.Fig.1

**A**

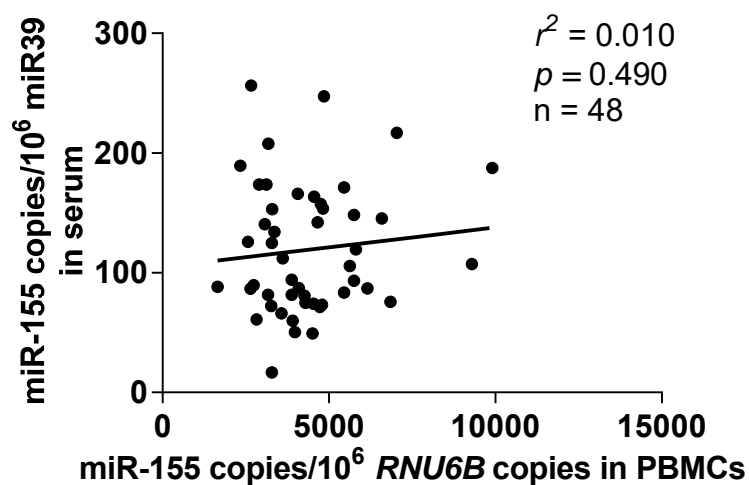

**B**

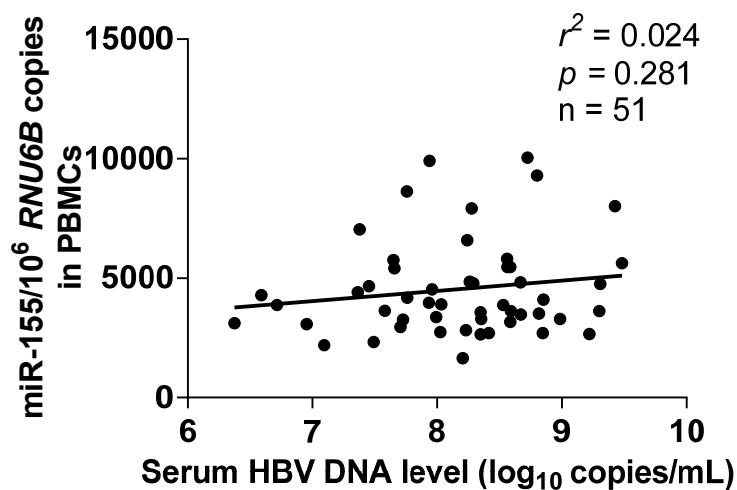

**C**

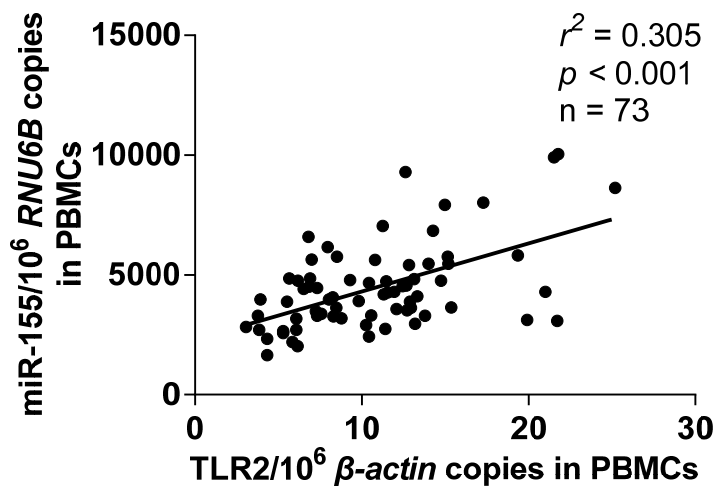

Suppl.Fig.2

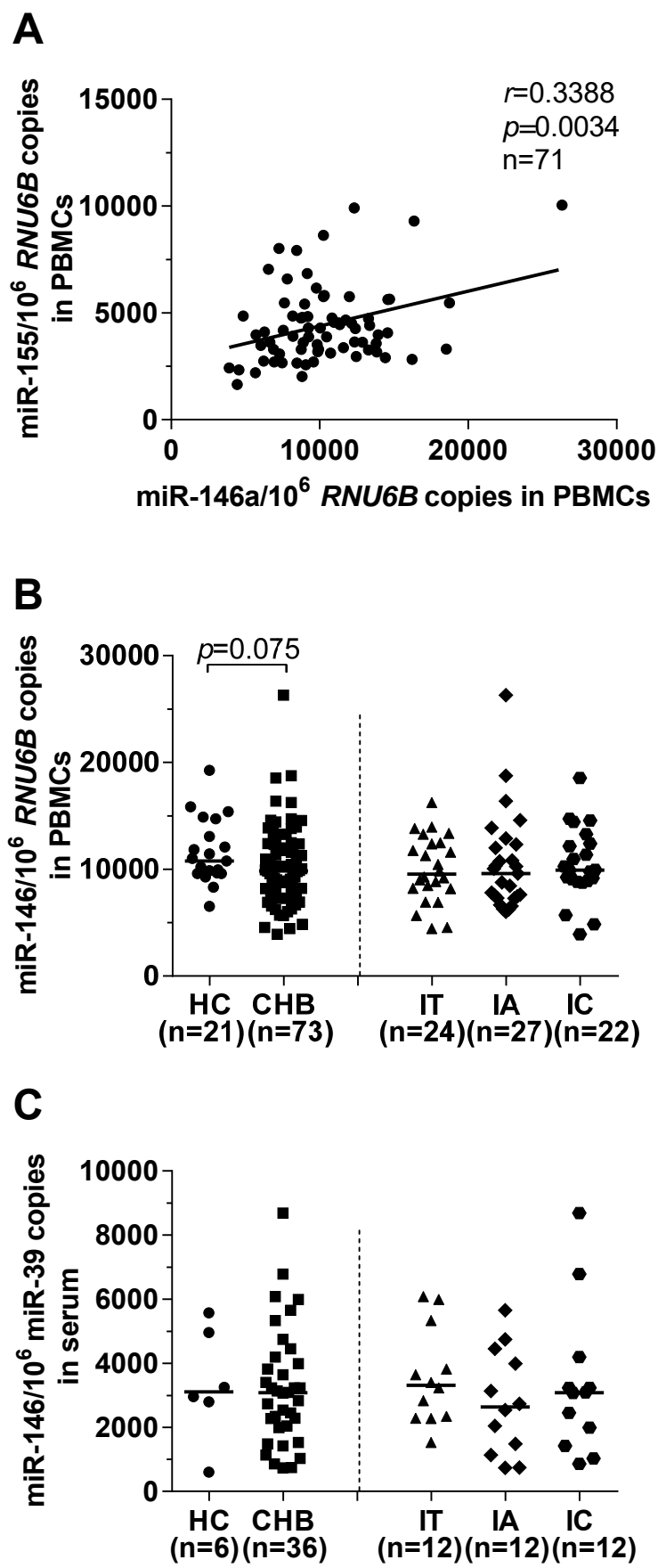

# Suppl.Fig.3

**A**

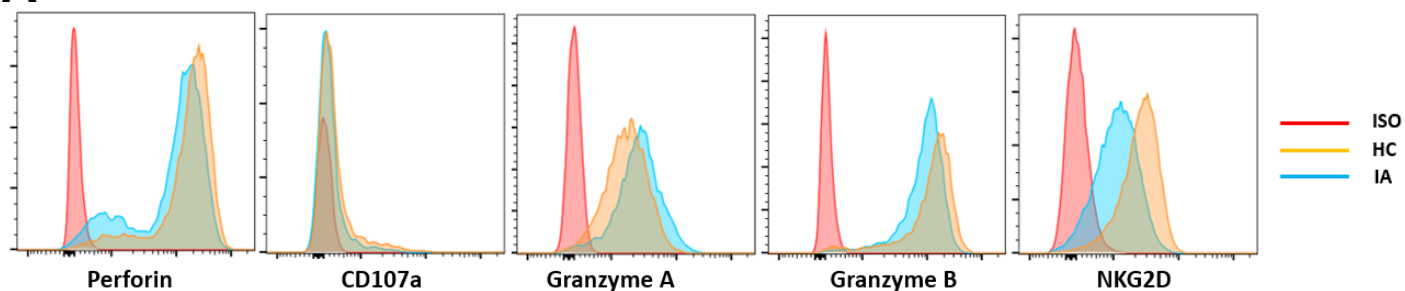

**B**

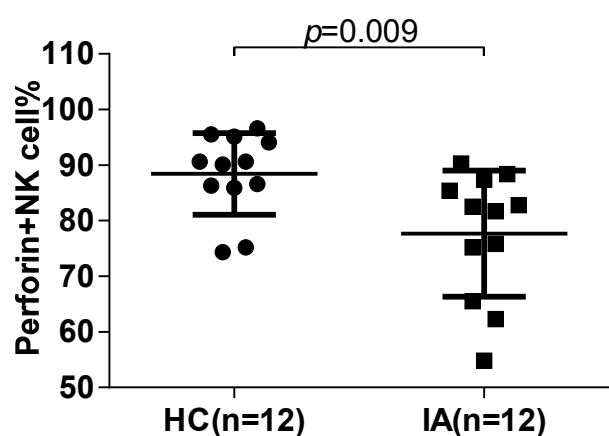

**C**

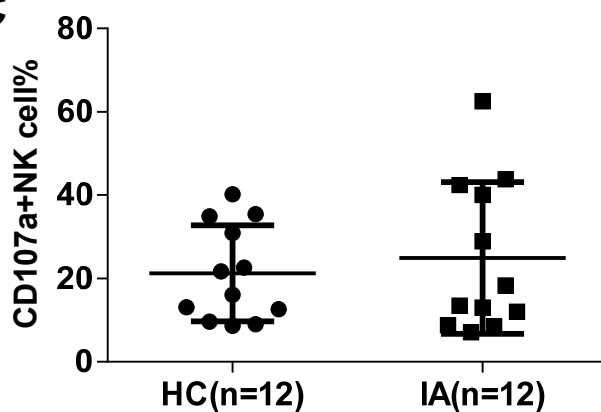

**D**

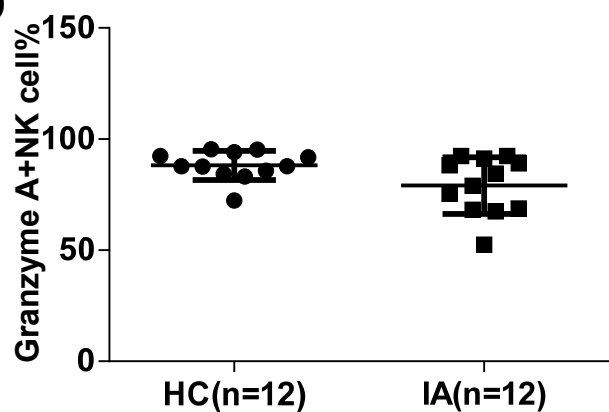

**E**

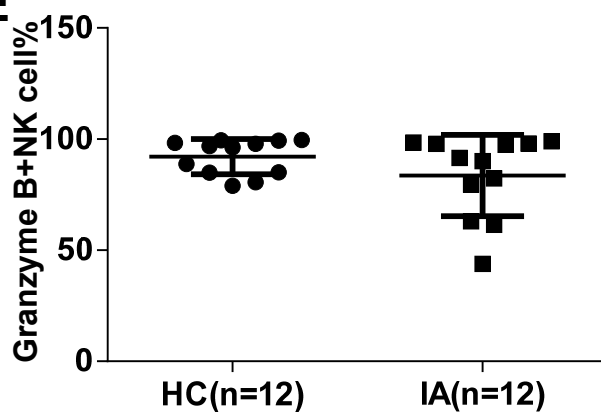

**F**

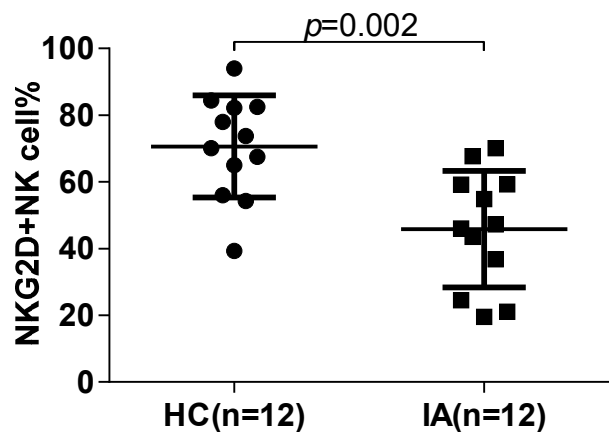

**G**

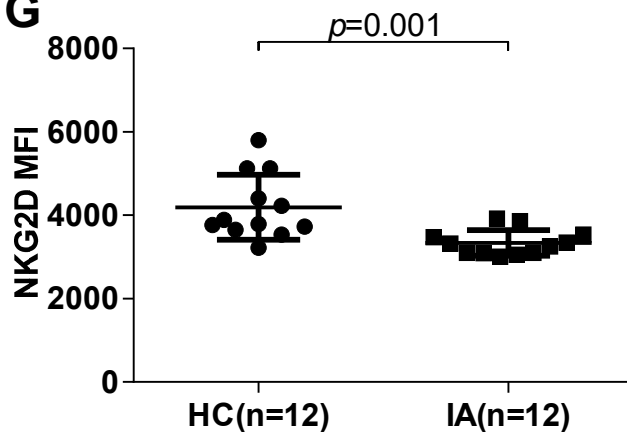

# Suppl.Fig.4

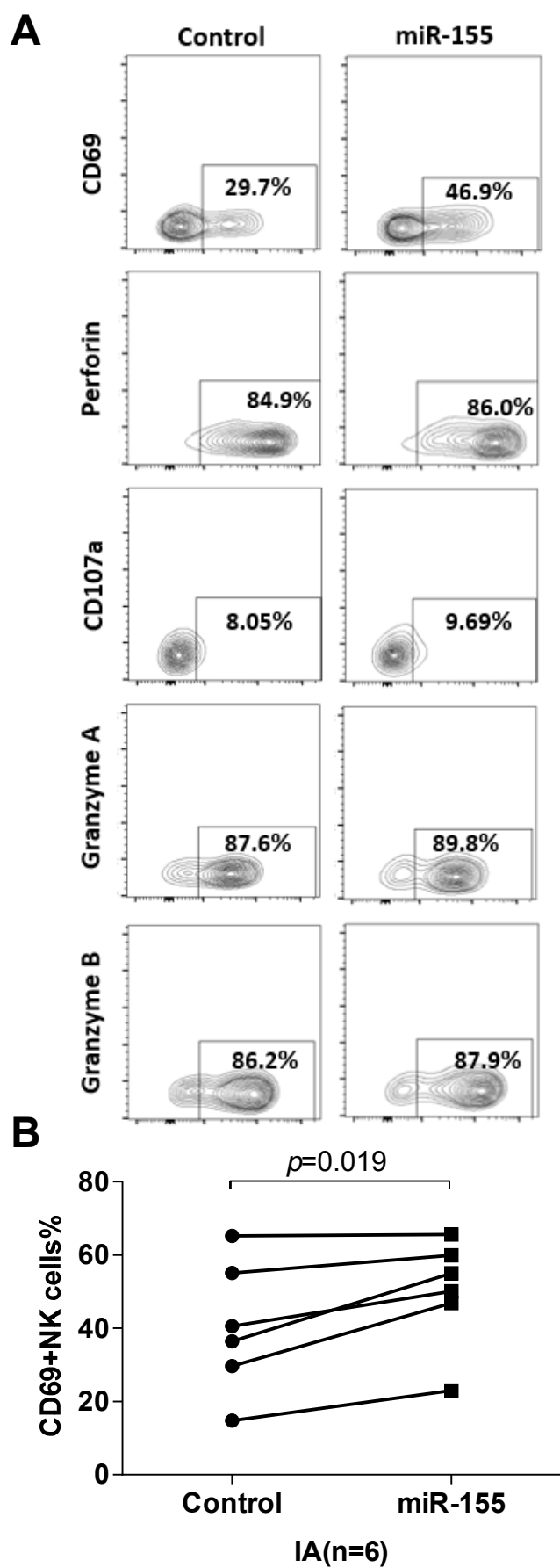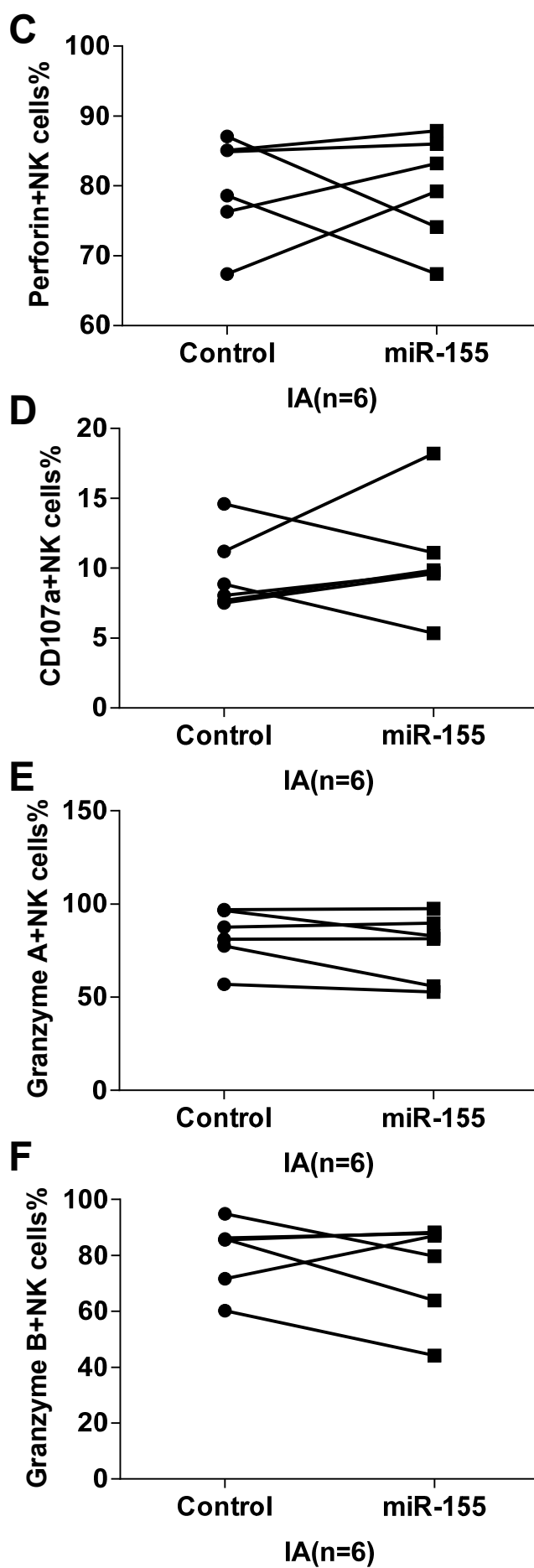

Suppl.Fig.4

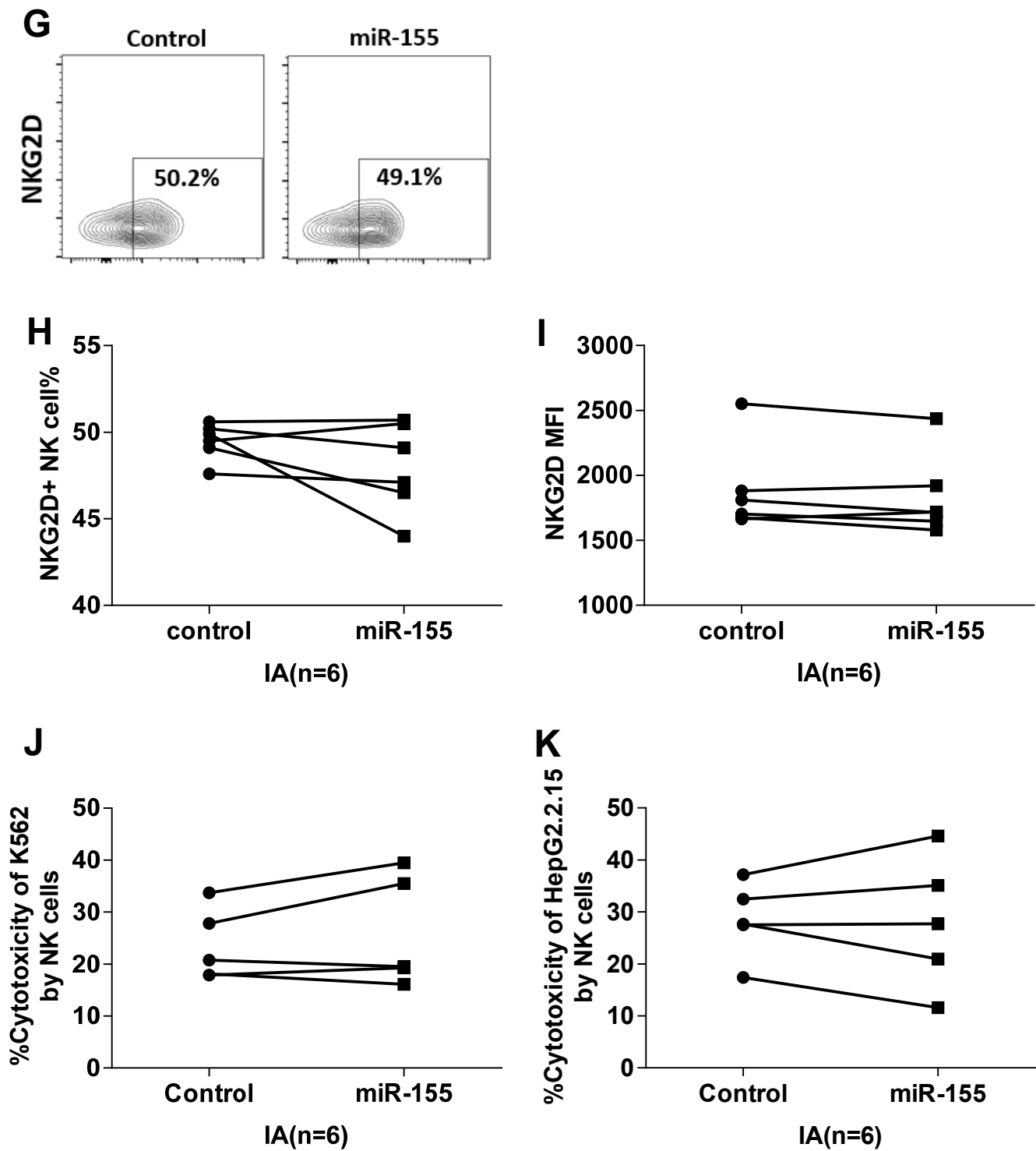

Suppl.Fig.5

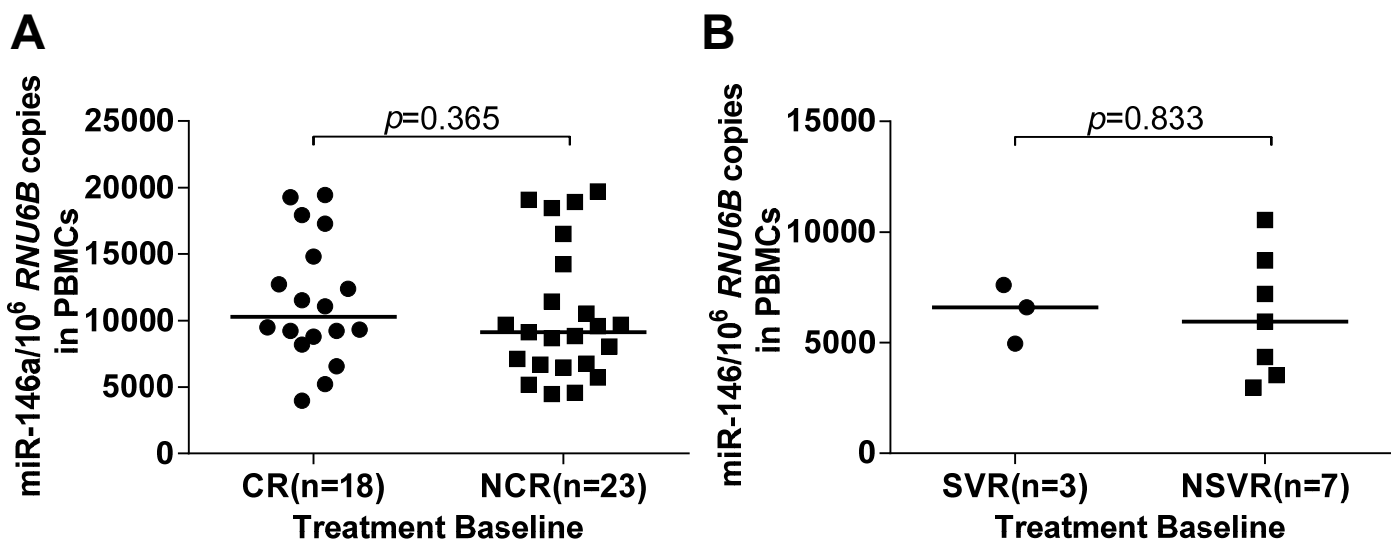

Supplement: Supplementary file 2 [file data_sheet_2.pdf]
